# Ketone-Assisted Alkoxysilane Condensation to Form Siloxane Bonds

**DOI:** 10.3390/molecules30143005

**Published:** 2025-07-17

**Authors:** Sławomir Rubinsztajn, Marek Cypryk, Jan Kurjata, Małgorzata Kwiatkowska, Urszula Mizerska

**Affiliations:** Centre of Molecular and Macromolecular Studies of Polish Academy of Sciences, Sienkiewicza 112, 90-363 Lodz, Poland; jan.kurjata@cbmm.lodz.pl (J.K.); malgorzata.kwiatkowska@cbmm.lodz.pl (M.K.); urszula.mizerska@cbmm.lodz.pl (U.M.)

**Keywords:** condensation, alkoxy-functional silanes, siloxane bond, ketone-assisted condensation, ketal, Ge(II) cationic complex

## Abstract

Siloxane bond formation represents a fundamental reaction central to both silicone chemistry and its technological applications. This paper presents a novel ketone-assisted process for the condensation of alkoxy-functional silanes catalyzed by a cationic Ge(II) complex stabilized by pentamethylcyclopentadiene Cp*Ge(II)^+^. This process leads to the formation of siloxane bonds, with dialkoxy ketal as a byproduct. Unlike the analogous reaction involving aldehydes, the ketone-assisted process is reversible, resulting in the formation of a mixture of alkoxy-functionalized silane or siloxane, along with the corresponding disiloxane product. Additionally, the introduced ketone underwent only partial conversion to the corresponding ketal. Furthermore, it was demonstrated that the siloxane bond could be cleaved to form alkoxysilane in the presence of the ketal and a cationic Cp*Ge(II) complex acting as a catalyst.

## 1. Introduction

We recently demonstrated that alkoxy-substituted silanes undergo a rapid and quantitative transformation into disiloxane and dialkyl acetal derivatives upon a reaction with stoichiometric amounts of aldehyde [1]. This novel condensation mechanism is catalyzed by a Ge(II)^+^ species, which is stabilized by pentamethylcyclopentadiene and paired with a weakly coordinating tetrakis(pentafluorophenyl)borate anion (Cp*Ge^+^ B(C_6_F_5_)_4_^−^).

We refer to this reaction as de-alkoxylation condensation. Our detailed mechanistic studies led us to conclude that the aldehyde-assisted de-alkoxylation condensation is a two-step process involving the formation of a mixed acetal as an intermediate.

Divalent cationic Si(II) and Ge(II) compounds stabilized by the pentamethylcyclopentadiene ligand were prepared for the first time by the team of Prof. Jutzi more than 20 years ago [2,3,4]. An excellent review of the research in this field has been published recently [5]. Wacker’s team prepared several new analogous compounds and demonstrated their effectiveness as catalysts in various important reactions, such as the hydrosilylation of a carbon–carbon double bond reaction, coupling between hydrosilanes and alkoxysilanes, and oxidative coupling of hydrosilanes in the presence of aldehydes and ketones [6,7,8].

The use of aldehydes to facilitate the curing of alkoxy-functional silicone resins has been described in recent patent applications by Wacker Chemie [9,10]. Our studies have shown that a disadvantage of this new condensation process is the need for stoichiometric amounts of aldehydes, such as propionaldehyde or acetaldehyde. Given that low-molecular-weight aldehydes are relatively toxic [11,12,13], replacing the aldehyde with a less toxic ketone, such as acetone [13], would make this reaction more acceptable for potential commercial applications. To determine whether ketones are indeed capable of enabling de-alkoxylation condensation, we performed a mechanistic study of the de-alkoxylation reaction in the presence of acetone.

In this article, we present our findings on the de-alkoxylation condensation of model alkoxy-functional silanes catalyzed by Cp*Ge^+^ B(C_6_F_5_)_4_^−^ in the presence of acetone. Additionally, we conducted further experiments to examine the de-alkoxylation reaction in the presence of acetophenone and benzophenone. To extend the scope of our study, we also investigated the cleavage of siloxane bonds by a corresponding ketal (2,2-dimethoxypropane) using a cationic Cp*Ge(II) complex as a catalyst.

## 2. Results

### 2.1. Study of the De-Alkoxylation Reaction in the Presence of Ketones

The de-alkoxylation reaction in the presence of acetone was investigated using model alkoxysilanes, such as PhMe_2_SiOMe and PhMe_2_SiOEt, in the presence of catalytic amounts of Cp*Ge^+^ B(C_6_F_5_)_4_^−^. ^1^H NMR and ^29^Si NMR spectroscopy, as well as GC and GC/MS, were used as the main analytical techniques to identify the substrate conversion and product formation.

**Figure 1:**
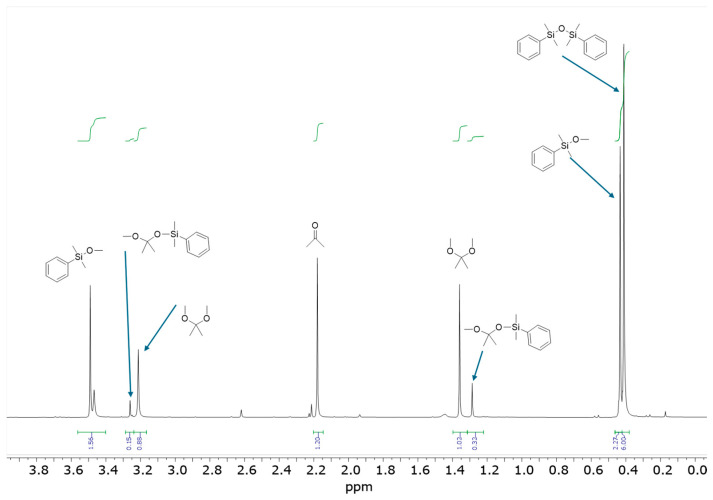
^1^H NMR spectrum of the reaction mixture that contained 2 equivalents of PhMe_2_SiOMe, 1 equivalent of acetone, and 0.02 equivalents of Cp*Ge^+^ B(C_6_F_5_)_4_^−^, as recorded after 24 h of reaction.

**Figure 2:**
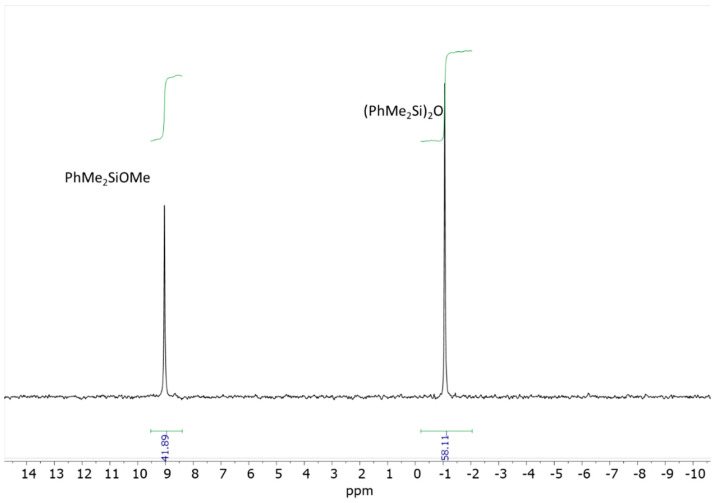
^29^Si NMR spectrum of the reaction mixture that contained 2 equivalents of PhMe_2_SiOMe, 1 equivalent of acetone, and 0.02 equivalents of Cp*Ge^+^ B(C_6_F_5_)_4_^−^, as recorded after 3 days of reaction.

The reaction between 2 equivalents of PhMe_2_SiOMe and 1 equivalent acetone in the presence of 0.02 equivalent of Cp*Ge^+^ B(C_6_F_5_)_4_^−^ did not result in the quantitative conversion of methoxy silane to the corresponding disiloxane, as previously observed in the analogous reaction with propionaldehyde [1]. The ^1^H NMR spectrum of the reaction mixture recorded after 24 h indicates an approximately 60% conversion of PhMe_2_SiOMe, as evidenced by two singlets at δ = 0.44 ppm (SiCH_3_) and 3.49 ppm (SiOCH_3_), to the corresponding disiloxane (singlet at δ = 0.42 ppm (SiCH_3_)). Additionally, acetone (singlet at δ = 2.17 ppm (CH_3_)) underwent a conversion to the 2,2-dimethoxypropane, as characterized by signals at δ = 1.36 ppm (CH_3_) and 3.22 ppm (OCH_3_) (Figure 1). Moreover, two other singlets observed at δ = 1.28 ppm (CH_3_) and δ = 3.26 ppm (OCH_3_) were assigned to a mixed ketal 2-methoxy-2-dimethylphenylsiloxypropane. The concentration of this mixed ketal was approximately 15 mol% relative to 2,2-dimethoxypropane. The presence of these two types of ketals was further confirmed by a GC/MS analysis of the reaction mixture (Figures S1 and S2). The ^29^Si NMR spectrum recorded after 3 days confirmed the presence of two major compounds: approximately 40 mol% of the starting PhMe_2_SiOMe (singlet at δ = 9.42 ppm) and the corresponding disiloxane (singlet at δ = −1.14 ppm) (Figure 2) [14,15].

**Figure 3:**
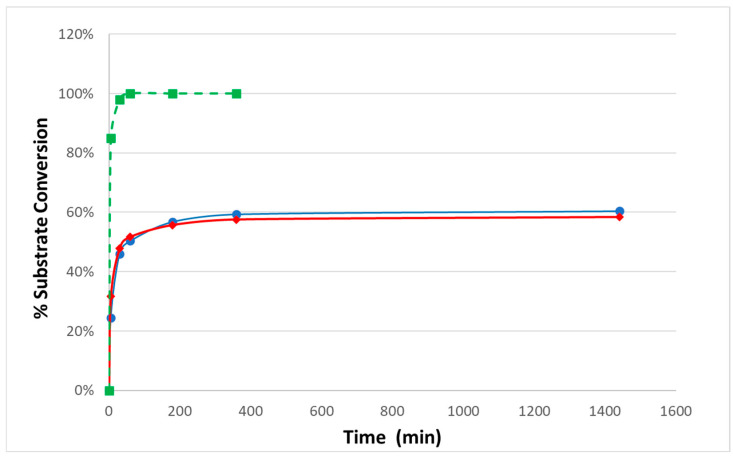
Comparison of alkoxysilane conversion over time in de-alkoxylation reactions: The green dashed line represents the conversion of 2 equivalents of PhMe_2_SiOMe in its reaction with 1 equivalent of propionaldehyde, catalyzed by 1 mol% of Cp*Ge^+^ B(C_6_F_5_)_4_^−^ [1]. The red solid line shows the PhMe_2_SiOMe conversion, while the blue solid line represents the acetone conversion in the de-alkoxylation reaction of 2 equivalents of PhMe_2_SiOMe with 1 equivalent of acetone, also catalyzed by 1 mol% of Cp*Ge^+^ B(C_6_F_5_)_4_^−^ in DCM.

The quantitative analysis of the reaction progress over time for the 2 equivalents of PhMe_2_SiOMe with 1 equivalent of acetone indicated that, indeed, the conversion of both reagents, PhMe_2_SiOMe and acetone, proceeded at the same rate. However, in the case of acetone, unlike the propionaldehyde [1], the de-alkoxylation process reached a plateau after about 6 h at approximately 60% conversion of methoxysilane (Figure 3). This phenomenon can be explained by the fact that the studied reaction in the presence of acetone is reversible and leads to equilibrium between reagents and products (Equation (1)). Based on the NMR data, the equilibrium constant for this process was estimated to be around 2.8.
(1)
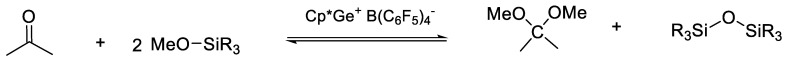


The reaction of the ethoxy-functional silane (PhMe_2_SiOEt) with acetone in the presence of 1 mol% Cp*Ge^+^ B(C_6_F_5_)_4_^−^ proceeded similarly to its methyl-functional counterpart. However, the conversion of PhMe_2_SiOEt to the corresponding disiloxane (diphenyltetramethyldisiloxane) proceeded more slowly, probably due to steric hindrance imposed by the ethyl group. Nevertheless, the steric effect of the ethyl group did not affect the equilibrium position between the alkoxysilane and the disiloxane. Like the reaction of PhMe_2_SiOMe with acetone, the conversion of PhMe_2_SiOEt to disiloxane reached about 60% at equilibrium, although over a longer period of about 48 h (Figure S3).

**Figure 4:**
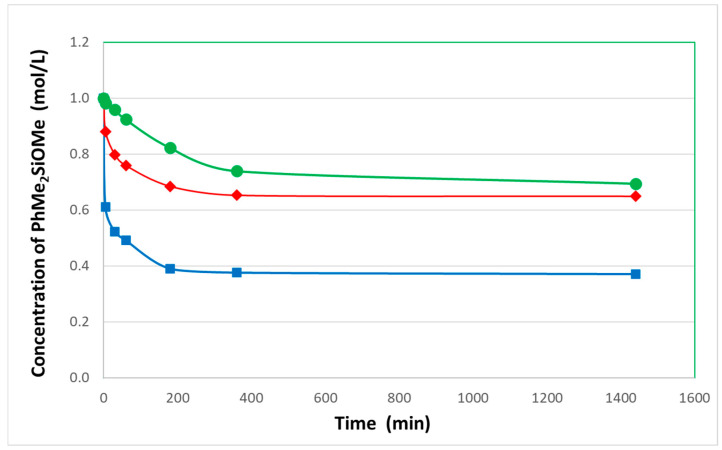
Effect of the ketone structure on the rate of PhMe_2_SiOMe conversion in the de-alkoxylation reactions in the presence of acetone (blue line), acetophenone (red line), and benzophenone (green line): [PhMe_2_SiOMe] = 1.0 mol/L and [Ketone] = 0.5 mol/L in the presence by 1 mol% of Cp*Ge^+^ B(C_6_F_5_)_4_^−^ in deuterated DCM.

The influence of the ketone structure on its reactivity in the de-alkoxylation reaction of PhMe_2_SiOMe was investigated for acetone, acetophenone, and benzophenone. Replacing a methyl group with a phenyl substituent significantly decreased the de-alkoxylation rate of the alkoxysilane (Figure 4). Furthermore, the phenyl-containing ketones exhibited a notably lower equilibrium conversion of alkoxysilane.

The observed effect of the phenyl substituent on the reactivity of the ketones in the series acetone, acetophenone, and benzophenone in the de-alkoxylation reaction can be explained by a combination of electronic and steric effects. The phenyl group contributes to resonance stabilization by delocalizing the electron density to the carbonyl system, thereby reducing the electrophilicity of the carbonyl carbon. As a result, ketones such as acetophenone and benzophenone exhibit lower reactivity compared with acetone. Moreover, the steric hindrance of the phenyl groups further inhibits nucleophilic attack, making the nucleophilic substitution at the carbonyl carbon in acetophenone and benzophenone slower than in acetone [16,17].

**Scheme 1:**
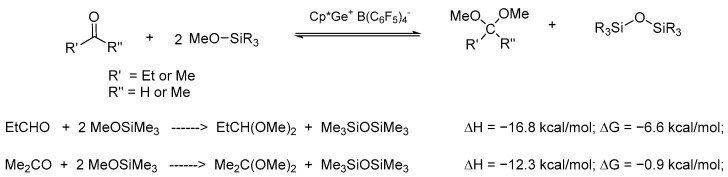
CBS-QB3 calculations for the de-alkoxylation reaction of Me_3_SiOMe in the presence of aldehyde and ketone in DCM.

Quantum chemical CBS-QB3 calculations for the de-alkoxylation reaction of Me_3_SiOMe in the presence of aldehydes and ketones in DCM revealed that the process of de-alkoxylation with an aldehyde exhibited a significantly negative Gibbs free energy change (ΔG = −6.6 kcal/mol), which corresponded to an equilibrium constant in the order of 10^4^–10^5^. In contrast, the analogous reaction with acetone had a higher ΔG of −0.9 kcal/mol, which corresponded to an equilibrium constant of approximately 4 (Scheme 1, Table 1).

**Scheme 2:**
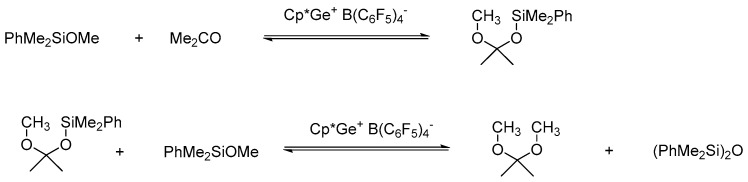
Proposed two-step de-alkoxylation condensation of alkoxysilanes in the presence of a stoichiometric amount of acetone.

These results align with experimental findings, confirming that the de-alkoxylation condensation of alkoxysilanes in the presence of acetone is a reversible process that establishes equilibrium between alkoxysilane and siloxane. They also support a two-step reversible mechanism for this reaction, involving the formation of a disiloxane and its corresponding dialkyl ketal, as illustrated in Scheme 2. The proposed mechanism of de-alkoxylation condensation is discussed in detail based on theoretical calculations in Section 2.4.

### 2.2. Reaction of Hexamethyldisiloxane with 2,2-Dimethoxypropane in the Presence of Cp*Ge^+^ B(C_6_F_5_)_4_^−^

**Figure 5:**
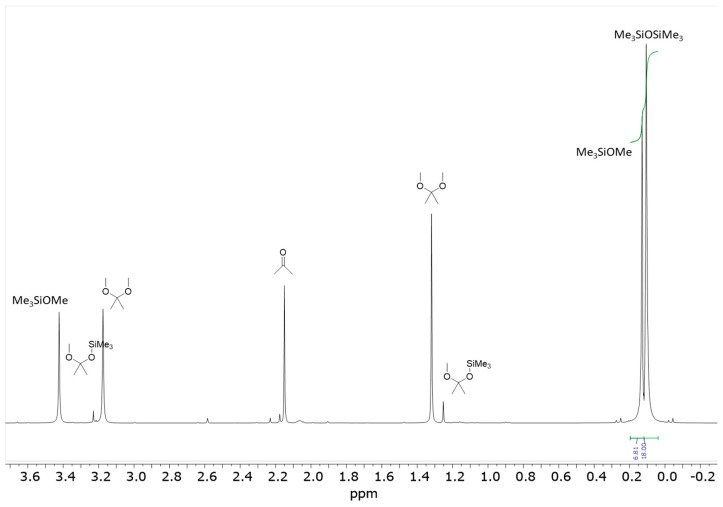
^1^H NMR spectrum of the reaction mixture that contained 1 mol/L of DMP, 1 mol/L of MM, and 0.01 mol/L of Cp*Ge^+^ B(C_6_F_5_)_4_^−^, as recorded after 24 h of reaction at RT.

**Figure 6:**
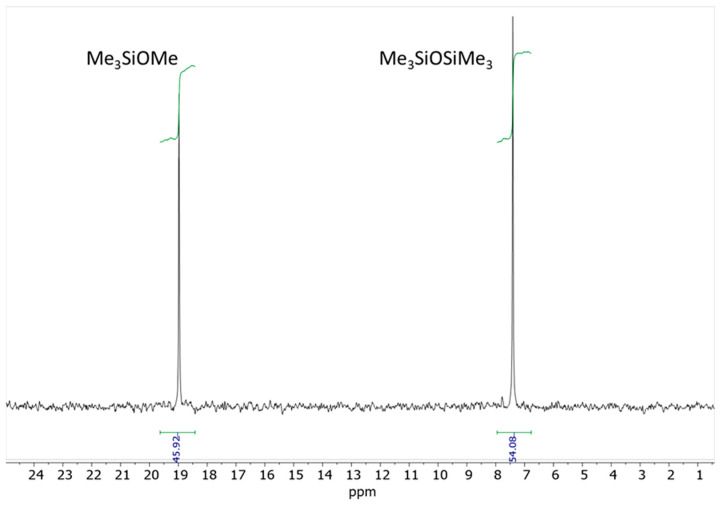
^29^Si NMR spectrum of the reaction mixture that contained 1 mol/L of DMP, 1 mol/L of MM, and 0.01 mol/L of Cp*Ge^+^ B(C_6_F_5_)_4_^−^, as recorded after 4 days of reaction at RT.

For the reaction of PhMe_2_SiOMe with acetone to be a reversible process, the resulting ketal (2,2-dimethoxypropane (DMP)) must undergo the reverse reaction with disiloxane. To prove this hypothesis, the reaction of 1 equivalent of DMP with 1 equivalent of hexamethyldisiloxane (MM) was investigated in the presence of 1 mol% of Cp*Ge^+^ B(C_6_F_5_)_4_^−^. After 24 h, when the reaction had reached equilibrium, the ^1^H NMR spectrum of the mixture revealed the presence of MM, Me_3_SiOMe, DMP, and acetone. Additionally, small amounts of a mixed ketal, (MeO)(Me_3_SiO)C(CH_3_)_2_, were detected (Figure 5). At this point, the conversion of DMP was approximately 44%. The ^29^Si NMR spectrum recorded after 4 days of reaction displayed two distinct singlets: one at δ = 7.22 ppm, which was assigned to MM, and another at δ = 18.87 ppm, which corresponded to the Me_3_SiOMe [14,15]. The integrated signal intensities indicate a molar ratio of Me_3_SiOMe to MM of approximately 0.85 to 1 (Figure 6).

**Scheme 3:**
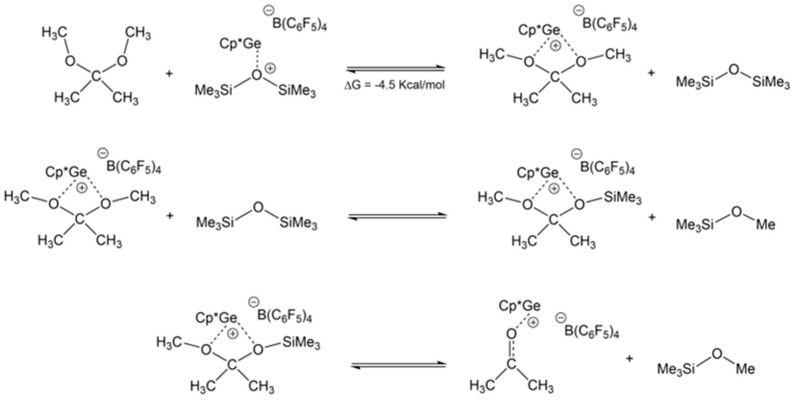
Siloxane bond cleavage in MM by DMP in the presence of Cp*Ge^+^ B(C_6_F_5_)_4_^−^.

The results above confirm that the de-alkoxylation reaction that involved the acetone was a reversible process and that DMP in the presence of Cp*Ge^+^ B(C_6_F_5_)_4_^−^ could break the siloxane bond. It must have been a two-step process (Scheme 3). Quantum chemical calculations indicated that most of the Cp*Ge^+^ cation interacted with DMP molecules in the MM and DMP mixture, with ΔG = −4.5 kcal/mol. In the thus formed Cp*Ge^+^-DMP complex, the ketal carbon of DMP was activated for nucleophilic attack by siloxane oxygen, leading to the formation of a mixed ketal and Me_3_SiOMe. The resulting mixed ketal underwent an intramolecular elimination reaction, which yielded acetone and an additional Me_3_SiOMe molecule (Scheme 3).

### 2.3. Reaction of Trimethylsilyl-Terminated Oligosiloxanes with 2,2-Dimethoxypropane (DMP) in the Presence of Cp*Ge^+^ B(C_6_F_5_)_4_^−^

**Scheme 4:**
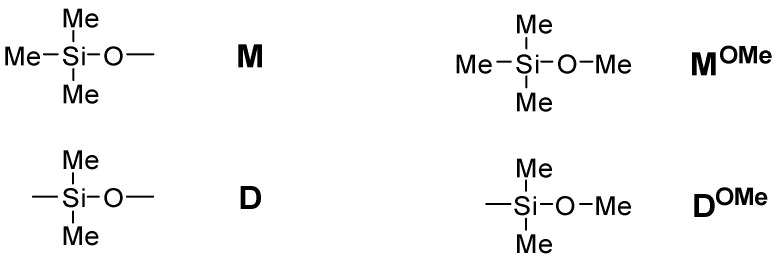
Shorthand notations of silicone units used in this manuscript.

To gain deeper insight into the cleavage of siloxane bonds by DMP, its reactions with a series of trimethylsilyl-terminated oligosiloxanes Me_3_SiO(SiMe_2_O)_x_SiMe_3_ (where x = 1, 2, 3, 4) were investigated in the presence of Cp*Ge^+^ B(C_6_F_5_)_4_^−^. In this manuscript, shorthand notations of siloxane oligomers were introduced to simplify their formulas, which are commonly used by silicone chemists (Scheme 4) [18]. Accordingly, Me_3_SiO(SiMe_2_O)_x_SiMe_3_ was abbreviated as MD_x_M.

**Figure 7:**
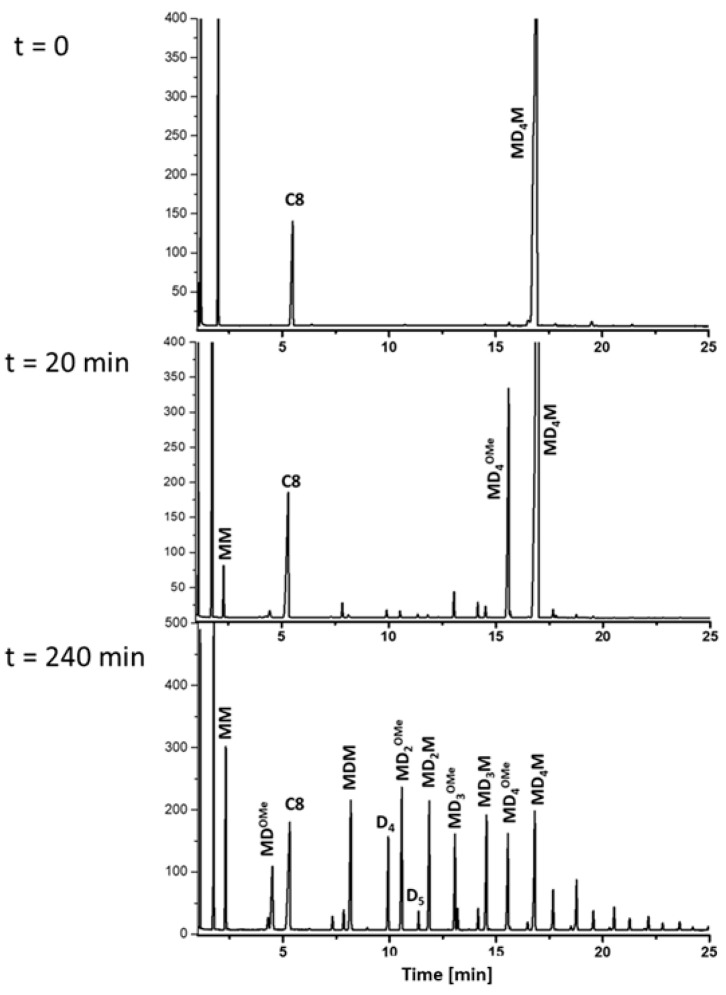
GC analysis of the reaction mixture of [MD_4_M] = 1.2 mol/L with [DMP] = 1.2 mol/L in the presence of [Cp*Ge^+^ B(C_6_F_5_)_4_^−^] = 0.0092 mol/L recorded at t = 0, t = 20 min, and t = 240 min.

**Figure 8:**
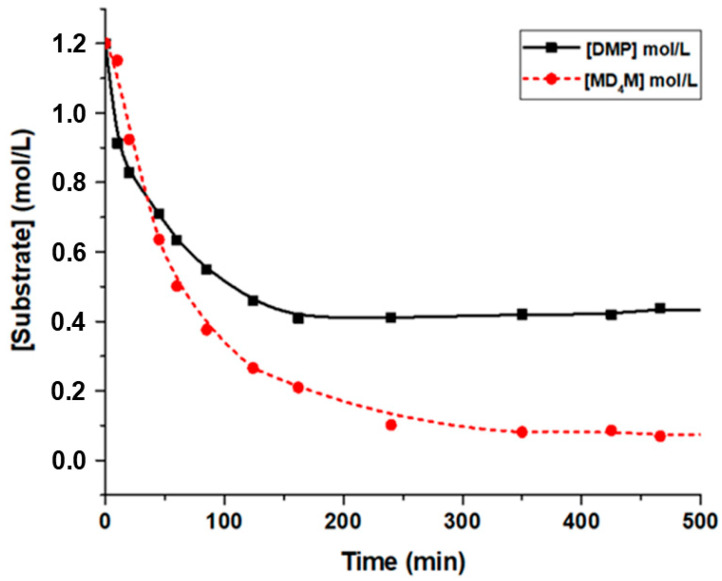
Conversion of MD_4_M (red dashed line) and DMP (black solid line) versus time in the reaction of [MD_4_M] = 1.2 mol/L with [DMP] = 1.2 mol/L in the presence of [Cp*Ge^+^ B(C_6_F_5_)_4_^−^] = 0.0092 mol/L.

**Figure 9:**
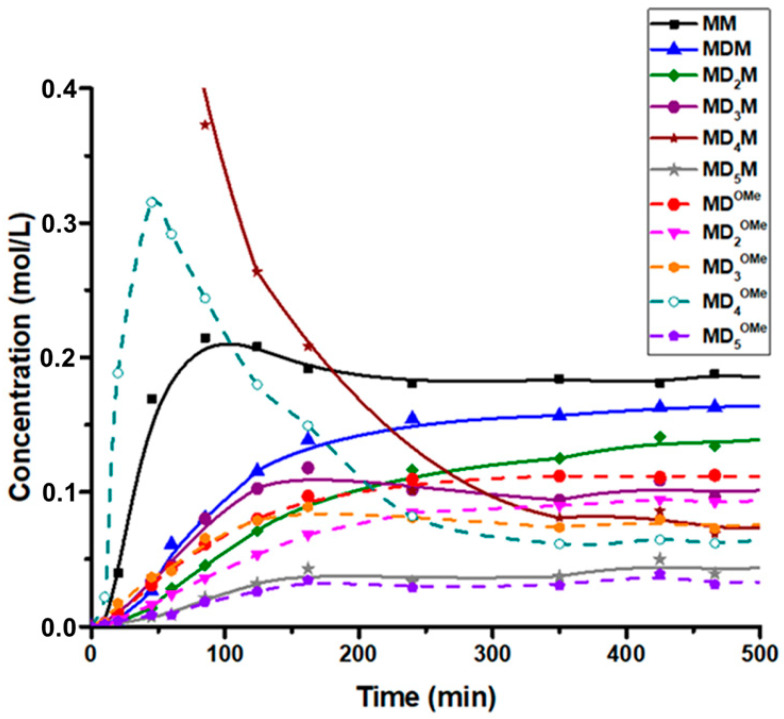
Substrate conversion and products formation versus time for the reaction of [MD_4_M] = 1.2 mol/L with [DMP] = 1.2 mol/L in the presence of [Cp*Ge^+^ B(C_6_F_5_)_4_^−^] = 0.0092 mol/L. Solid lines represent MDXM oligomers; dashed lines represent MD_x_^OMe^ oligomers.

**Figure 10:**
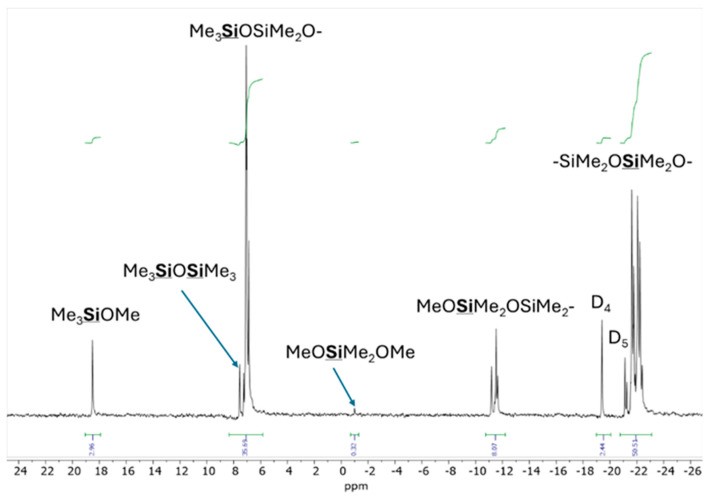
^29^Si NMR of the reaction mixture that contained [MD_4_M] = 1.2 mol/L and [DMP] = 1.2 mol/L in the presence of [Cp*Ge^+^ B(C_6_F_5_)_4_^−^] = 0.0092 mol/L, as recorded after 7 days of reaction at RT.

**Scheme 5:**
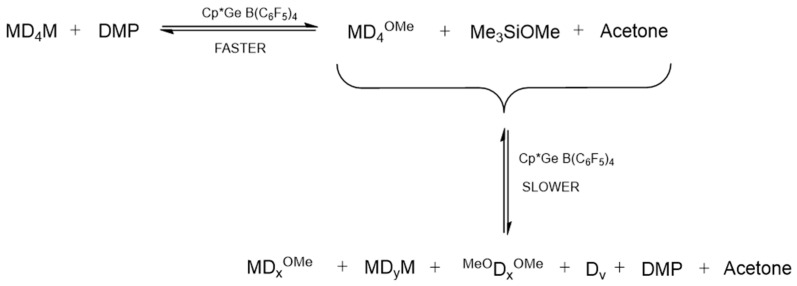
Reactions observed in the system MD_4_M + DMP in the presence of catalytic amount of Cp*Ge^+^ B(C_6_F_5_)_4_^−^.

In the first experiment, the reaction of Me_3_SiO(SiMe_2_O)_4_SiMe_3_ (MD_4_M) with an equimolar concentration of DMP in the presence of 0.8 mol% of Cp*Ge^+^ B(C_6_F_5_)_4_^−^ was studied. The conversion of MD_4_M and DMP and the formation of oligomeric siloxanes were monitored by GC (Figure 7). The chemical identity of the observed peaks was confirmed in a separate GC/MS experiment (Figure S4). The process occurred in two distinct stages. During the first stage, the conversion rates of both reagents were approximately similar. However, after approximately 60 min, the conversion rate of DMP slowed significantly, where they eventually reached a plateau at around 0.5 mol/L (60% conversion) after 200 min. Meanwhile, the concentration of MD_4_M continued to decrease until it stabilizes at an equilibrium concentration of approximately 0.1 mol/L (Figure 8). Interestingly, under these conditions, the preferential formation of MD_4_^OMe^ and Me_3_SiOSiMe_3_ (MM) was observed during the initial stage of the process (Figure 9). These results indicate that the cleavage of the terminal siloxane bond in MD_4_M by DMP, which led to the formation of MD_4_^OMe^, Me_3_SiOMe (M^OMe^), and acetone, was the fastest reaction in this system. The resulting M^OMe^ appeared to be highly reactive under these conditions and rapidly underwent acetone-assisted condensation to form MM and other M-stopped siloxanes. However, after reaching the maximum concentration at about 60 min of reaction, the concentration of the monoalkoxy oligosiloxane (MD_4_^OMe^) started to decline. This process was accompanied by the formation of various oligomeric alkoxy-functional and M-stopped siloxanes. The reaction observed seemed to reach an equilibrium after about 8 h. The predominant products were two series of linear oligosiloxanes, MD_x_M and MD_x_^OMe^. Additionally, cyclic siloxanes (mostly D_4_ and D_5_) and dimethoxy-stopped oligosiloxanes (^MeO^D_x_^OMe^) were formed, though their concentrations remained lower. The ^29^Si NMR spectrum recorded after 7 days confirmed the presence of several products (Figure 10). The singlet at δ = 18.53 ppm was assigned to Me_3_SiOMe, while the singlet at δ = 7.55 ppm corresponded to Me_3_SiOSiMe_3_. An unresolved multiplet centered at δ = 7.10 ppm was attributed to the Me_3_SiO groups connected to various oligosiloxanes, whereas another unresolved multiplet at δ = −11.4 ppm was associated with the MeOSiMe_2_ groups within different oligosiloxanes. Additionally, distinct singlets at δ = −19.4 ppm and δ = −21.11 ppm were assigned to cyclic D_4_ and D_5_, respectively, and an unresolved multiplet centered at δ = −21.8 ppm corresponded to various -SiMe_2_O- units [15]. The formation of various MD_x_M, MD_x_^OMe^, and cyclic oligosiloxanes can be explained by a siloxane bond disproportionation process, which was also catalyzed by Cp*Ge^+^ B(C_6_F_5_)_4_^−^ (Scheme 5).

**Scheme 6:**
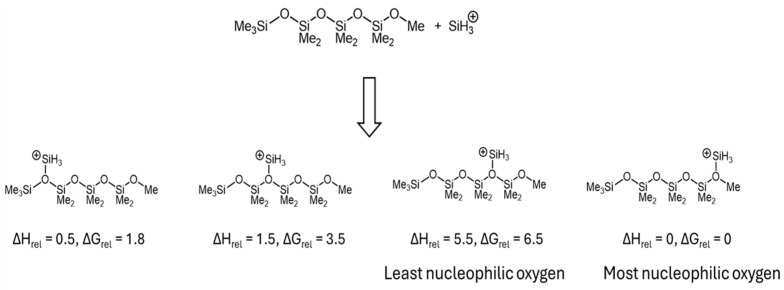
Relative DFT enthalpies and Gibbs free energies (kcal/mol) for the hypothetical complexes between the linear alkoxy-functional siloxane Me_3_Si(OSiMe_2_)_3_OMe and SiH_3_^+^.

Thus, in the studied reaction of DMP with MD_4_M catalyzed by Cp*Ge^+^ B(C_6_F_5_)_4_^−^, a fast cleavage reaction of the terminal siloxane bond by DMP took place, which led to the formation of monoalkoxy oligosiloxane MD_4_^OMe^. To explain this phenomenon, the relative nucleophilicities of oxygen atoms in the short trimethylsilyl-terminated PDMS chains (Me_3_Si(OSiMe_2_)_3_OMe) were evaluated by DFT calculations using SiH_3_^+^ as the reference Lewis acid (Scheme 6).

The density function theory (DFT) calculations revealed that the relative nucleophilicities of oxygen atoms in short linear oligosiloxanes of the formula Me_3_Si(OSiMe_2_)_x_OMe (MD_x_^OMe^) were not equal. The oxygen adjacent to the terminal Me_3_Si group exhibited higher nucleophilicity compared with the oxygen atom bonded to two SiMe_2_O units (Scheme 6). Similarly, in MD_4_M, the oxygen atom linked to the terminal trimethylsilyl group was expected to be the most nucleophilic in the nucleophilic substitution reaction at the activated carbon center of DMP, which was complexed by Cp*Ge^+^ B(C_6_F_5_)_4_^−^. This nucleophilic attack was anticipated to result in cleavage of the siloxane bond, which led to the formation of MD_4_^OMe^, Me_3_SiOMe, and acetone, as illustrated in Scheme 5. The enhanced reactivity of the terminal (M-stopped) siloxane bond was previously observed in the cationic oligomerization of linear siloxanes [19]. The resulting Me_3_SiOMe was likely the most reactive alkoxy-functional compound, where it underwent rapid conversion to MM via a de-alkoxylation process in the presence of acetone, as experimentally observed (Figure 9). However, even at equilibrium, a significant amount of Me_3_SiOMe remained in the reaction mixture (Figure 10). The MD_4_^OMe^ oligomer, initially formed in excess, subsequently underwent siloxane bond disproportionation reactions and acetone assisted de-alkoxylation condensation, which led to the formation of various linear siloxane oligomers with the general formulas MD_x_M and MD_x_^OMe^ (Scheme 5).

The scope of these studies was further expanded to investigate the reactions between DMP and other M-stopped short linear siloxanes, including MDM, MD_2_M, and MD_3_M. The reactions were carried out at a 1:1 molar ratio of DMP to MD_x_M, where x = 1, 2, 3, and 1 mol% of Cp*Ge^+^ B(C_6_F_5_)_4_^−^ served as the catalyst. These transformations followed the same pathway as the reaction between DMP and MD_4_M, as illustrated in Figures S5–S7. The cleavage of the terminal siloxane bond was the fastest step in these reactions, irrespective of the number of D units. This process resulted in the formation of excess MD^OMe^ in the reaction with MDM, MD_2_^OMe^ in the reaction with MD_2_M, and MD_3_^OMe^ in the reaction with MD_3_M. As previously observed in the reaction of DMP with MD_4_M, the concentration of the initially formed in excess mono-alkoxy siloxane gradually decreased as the reaction progressed. Meanwhile, other M-stopped and alkoxy-functional siloxanes emerged and eventually reached equilibrium concentrations after several hours.

### 2.4. Mechanistic Study of Ketone-Assisted De-Alkoxylation via DFT and CBS-QB3 Calculations

The experimental findings support the proposed mechanism for the de-alkoxylation reaction in the presence of a ketone, as outlined in Scheme 2. To further elucidate this process, DFT calculations were performed. It seemed reasonable to assume that the mechanism of de-alkoxylation involving ketones would closely resemble that reported for aldehydes [1]. Therefore, the geometries of stationary points found for the reaction with propionaldehyde, after necessary modification, were used as starting points for searching the stationary structures in the reaction with acetone. To simplify the DFT calculations, the presence of the weakly nucleophilic anion B(C_6_F_5_)_4_^−^ was omitted. For the same reason, the methyl groups in the cyclopentadiene ligand were replaced by hydrogens. However, a comparison between the DFT calculations and experimental data revealed a significant discrepancy. The DFT calculations predicted the Gibbs free energy change (ΔG) for the acetone-assisted de-alkoxylation reaction of PhMe_2_SiOMe to be approximately 5.6 kcal/mol, which corresponded to an equilibrium constant of about 10^−4^. However, experimental results indicate a much higher equilibrium constant of approximately 2.8, corresponding to a ΔG of ca. −0.6 kcal/mol.

To verify the DFT calculation results, the CBS-QB3 composite method was employed to recalculate the thermochemistry for key reactions and equilibria occurring in the reaction system. This robust and cost-effective approach offers high accuracy across various chemical applications (often within 1–2 kcal/mol of experimental values) by integrating multiple calculation steps and empirical corrections [20]. The comparison of the results obtained using the DFT and CBS-QB3 methods are presented in Table 1.

The above results show that the DFT calculations were in some cases (for example, for the thermodynamics of the overall reactions with EtCHO (reaction A) and Me_2_CO (reaction B)) significantly less accurate than the CBS-QB3 calculations. Thus, when the thermochemistry of chemical equilibria is discussed, we usually refer to the CBS-QB3 results. Furthermore, the comparison of the CBS-QB3 results for the gas phase and for the DCM solution showed that the solvent effect could be estimated as 1–2.5 kcal/mol. To most accurately represent the real reaction system, we used the values obtained for the reaction in solution. However, the free energy profile for the proposed condensation mechanism was calculated by the DFT method for two reasons. First, CBS-QB3 calculations are significantly more computationally expensive; second, we aimed to preserve compatibility with our previous report to enable a direct comparison between aldehyde and ketone.

The DFT calculations for the gas-phase conditions indicate that CpGe^+^ interacts more strongly with methoxy silane than with propionaldehyde, which yielded an equilibrium constant (K_eq_) of 2.7 for reaction C (Table 1). The CBS-QB3 method predicted an even larger K_eq_ for this equilibrium in DCM. Given that acetone is a stronger nucleophile than propionaldehyde due to the electron-donating effects of its two methyl groups, one would expect the equilibrium of reaction D (Table 1) to shift toward the CpGe^+^–acetone complex. Indeed, the gas-phase DFT calculations confirmed this expectation, where they yielded a K_eq_ value of 0.2. Consequently, the concentration of the CpGe^+^–acetone complex was approximately 5 times higher than that of the CpGe^+^ complex with Me_3_SiOMe. However, the CBS-QB3 method predicted a significantly larger K_eq_ value of 7 for this system in DCM, indicating that only approximately 30 mol% of CpGe^+^ interacted with acetone. It seems that the energy of the CpGe^+^–acetone interaction was somewhat underestimated due either to the deficiency of the CBS-QB3 method itself or to inaccuracy in the solvent effect estimation.

**Scheme 7:**
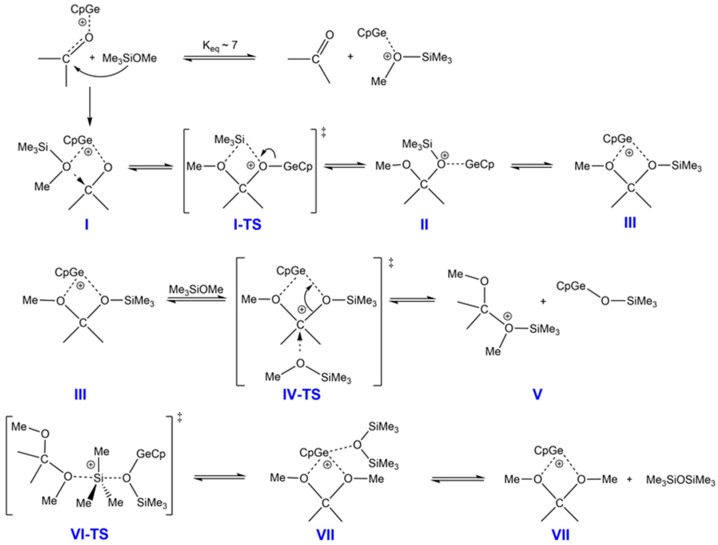
Proposed mechanism of the ketone-assisted de-alkoxylation reaction in the presence of CpGe^+^ catalyst.

Based on the DFT results for reaction D, further investigation was conducted into the mechanism of the de-alkoxylation process. This reaction follows a widely accepted mechanism in the literature in which a nucleophile—specifically an alkoxy silane—attacks the carbon center of acetone [17], which is activated by its interaction with the cationic CpGe(II)^+^ complex (Scheme 7).

**Figure 11:**
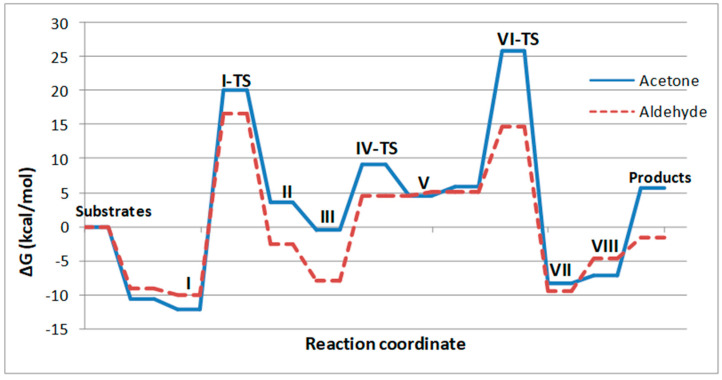
DFT-calculated Gibbs free energy profile for the reaction between Me_3_SiOMe and Me_2_CO catalyzed by CpGe^+^ in the gas phase. For comparison, the profile for the reaction of Me_3_SiOMe + EtCHO was reproduced from our previous publication [1].

The initial condensation step involves the nucleophilic attack of methoxysilane on the carbonyl carbon, which is activated by a germanium cation (I). This species is in equilibrium with a complex of methoxysilane and the CpGe^+^ catalyst. Although this latter complex is more abundant, calculations suggest that the attack of acetone on silicon, when activated by the germanium cation, involves a higher energy barrier. Consequently, the silyl group is transferred to the carbonyl oxygen (forming intermediate II) leading to the formation of a mixed methoxy–siloxy ketal (intermediate III). Subsequently, another methoxy silane molecule attacks, substituting for the trimethylsiloxy–germanium intermediate (V). This intermediate then undergoes a rearrangement, leading to the cleavage of a trimethylsilyl group and the formation of the Ge mixed complex (VII), followed by the final formation of disiloxane and the germanium–DMP complex (VIII). The corresponding Gibbs free energy profile for the stationary points on the reaction pathway is presented in Figure 11 and the corresponding relative enthalpies and Gibbs free energies are shown in Table 2. The calculated structures of the stationary points and transition states are presented in Figure S8. The formation of the CpGe^+^–DMP complex was further supported by a ^1^H NMR experiment. The ^1^H NMR spectrum of a reaction mixture that contained 1 equivalent of CpGe^+^ B(C_6_F_5_)_4_^−^ and 100 equivalents of DMP exhibited a slight upfield chemical shift of the methyl protons on the Cp* ring, which is consistent with complex formation (Figure S9).

It is apparent that the de-alkoxylation reaction with aldehyde exhibited lower energy barriers than the reaction with ketone and, consequently, was predicted to be considerably faster (Figure 11). It was also more thermodynamically favorable (see the CBS-QB3 values for a more precise evaluation), which confirmed that the reaction proceeded practically to completion, in contrast to the reaction with ketone.

## 3. Materials and Methods

### 3.1. Materials

Phenyldimethylsilane (Fluorochem, 98%) and 2,2-dimethoxypropane (Merck KGaA, Darmstadt, Germany, 97%), were used without further purification. Acetone (Merck KGaA, Darmstadt, Germany, 97%) was dried and purified by distillation. Pd/C (5%) was purchased from Merck KGaA, Darmstadt, Germany. Hexamethyldisiloxane, MDM, MD_2_M, MD_3_M, and MD_4_M were purchased from ABCR GmbH, Karlsruhe, Germany. Cp*Ge^+^ B(C_6_F_5_)_4_^−^ catalyst was obtained from Wacker Chemie AG, Munich, Germany and was used without further purification. All solvents—DCM, Et_2_O, hexane, and toluene—were dried using standard procedures.

### 3.2. Synthesis Alkoxysilanes and Siloxanes

Model alkoxy-functional silanes and siloxanes were synthesized using a similar procedure to that outlined below for dimethylphenylethoxysilane (PhMe_2_SiOEt). In a round-bottomed flask equipped with a reflux condenser and addition funnel, 8.89 g (0.064 mol) of PhMe_2_SiH, 8 mL of hexane, and 0.131 g of 5% Pd/C were introduced. The reaction was conducted under an inert argon atmosphere. Subsequently, 7.24 g (0.156 mol) of EtOH in 7 mL of hexane was added dropwise to the reaction mixture. After 15 min, GC analysis confirmed complete conversion of the silane. The mixture was then filtered through celite to remove the catalyst, and hexane was evaporated under vacuum, which yielded 8.28 g (74.02%) of crude product. The GC analysis verified the formation of PhMe_2_SiOEt and the complete conversion of the substrate. The crude reaction product was then distilled using a small Vigreux column, which yielded 7.95 g of PhMe_2_SiOEt with approximately 95% purity (GC), at a boiling point of 180 °C/15 mmHg. ^1^HNMR (400 MHz, CDCl_3_): δ = 0.41 ppm (s, 6H), δ = 1.06 ppm (s, 3H, OCH_3_), δ = 7.42 ppm (m, 3H, para and meta), δ = 7.63 ppm (m, 2H, CH ortho). ^29^Si NMR (79.46 MHz, CDCl_3_): δ = 9.04 ppm (s, SiOMe).

### 3.3. NMR Spectroscopy

^1^H and ^29^Si NMR spectra in CDCl_3_ and CD_2_Cl_2_ were obtained with a Bruker, Billerica, MA, USA 400 MHz spectrometer operated at 400 and 79.46 MHz, respectively. The ^29^Si NMR spectra were recorded with broadband proton decoupling. Heteronuclear gated decoupling with a 20 s delay technique was used to acquire the ^29^Si NMR spectra.

### 3.4. Gas Chromatography–Mass Spectrometry (GC/MS)

GC/MS analysis was performed using a Shimadzu, Kyoto, Japan, QP2010 ultra-apparatus equipped with a Zebron ZB-5MSi Capillary GC Column (30 m × 0.25 mm × 0.25 μm), Phenomenex, Torrance, CA, USA. The carrier gas was helium. The following temperature program was used: hold at 50 °C for 3 min, heat to 250 °C at a rate of 10 °C/min, hold at 250 °C for 20 min, and heat to 280 °C at a rate of 20 °C/min. The quadrupole mass spectrometer, Shimadzu QP2010 Ultra, with electron ionization was connected to a GC system.

### 3.5. Gas Chromatography

Gas chromatography analyses were performed using an Agilent 8860 GC System (Agilent Technologies, Inc., Santa Clara, CA, USA) equipped with a thermal conductivity detector and HP-1 capillary column (Agilent Technologies, Inc., Santa Clara, CA, USA) with diameter 0.53 mm and length 30 m. The carrier gas was helium, with flow rate 10 mL/min, detector temperature 250 °C, injector temperature 250 °C, and the following column temperature program: hold 5 min at a 40 °C isotherm, heat to 240 °C at a rate of 10 °C/min and hold at 240 °C for 10 min.

### 3.6. Study of the De-Alkoxylation of Model Alkoxysilanes in the Presence of Acetone

In a typical experiment, 58 mg (2 equivalents) of PhMe_2_SiOMe and 10.1 mg (1 equivalent) of acetone were placed in an NMR tube along with 500 µL of deuterated DCM. The first ^1^H NMR spectrum was recorded. Subsequently, 0.02 equivalents of Cp*Ge^+^ B(C_6_F_5_)_4_^−^, dissolved in DCM, was added, and a second ^1^H NMR spectrum was collected approximately 5 min later. Additional ^1^H NMR spectra were acquired at time intervals of 30 min, 1 h, 3 h, 6 h, 24 h, and 48 h. In some experiments, extra samples were taken and analyzed by GC-MS. The final sample was analyzed by ^29^Si NMR. ^29^Si NMR (79.46 MHz, CDCl_3_): δ = 9.04 ppm (s, SiOMe), δ = −1.05 ppm (s, SiOSi).

### 3.7. Study of the Reaction MD_x_M + DMP, Where x = 0, 1, 2, 3, 4

In a typical experiment, 0.446 g (1.0 mmol) of MD_4_M, 0.5 g (0.32 mL) of dried DCM, and 0.035 g (0.31 mmol) of octane (used as an internal standard) were placed in a small glass vial equipped with a magnetic stirrer and a rubber septum. A time-zero sample was withdrawn through the septum using a hypodermic syringe and analyzed by GC. Next, 100 µL of a 0.05 mol/L solution of Cp*Ge^+^ B(C_6_F_5_)_4_^−^ (0.01 mmol) in DCM was introduced into the stirred solution at 25 °C via a syringe through the septum, marking the start of the reaction. Samples of the reaction mixture were then withdrawn at specified time intervals using a hypodermic syringe and subjected to GC analysis equipped with a TCD detector. The concentrations of MD_x_M and other siloxane oligomers were determined using the internal standard method with n-octane. The retention times and the chemical identity of the resulting siloxane oligomers were determined by GC-MS. Their concentrations were analyzed using the internal standard method with n-octane. Molar response factors for the siloxane oligomers were established in separate experiments using MDₓM solutions of known composition. The final sample was analyzed by ^29^Si NMR. ^29^Si NMR (79.46 MHz, CDCl_3_): δ = 18.48 ppm (s, Me_3_SiOMe), δ = 7.54 ppm (s, MM), δ = 7.2 ppm (m, Me_3_Si-O-), δ = −1.05 ppm (s, Me_2_Si(OMe)_2_), δ = −11.43 ppm (m, MeO-SiMe_2_-O-), δ = −19.41 ppm (s, (Me_2_SiO)_4_), δ = −21.11 ppm (s, (Me_2_SiO)_5_), δ = −21.91 ppm (m, -O-SiMe_2_-O-).

### 3.8. Theoretical Methods

All electronic structure calculations in this work were carried out using the Gaussian 16 software package [21]. The geometries of the bases and base pair model systems were optimized using the hybrid B3LYP density functional [22] corrected for dispersion interactions using the Grimme D3 empirical term with Becke–Johnson damping [23,24], with the Def2TZVP basis set in the gas phase. This level of theory is denoted as B3LYP-D3BJ/Def2TZVP. The Def2xxVP family of basis sets was chosen because it covers a wide range of elements up to Rn and was proved to give consistent and reliable results [25]. All stationary points were identified as stable minima or transition states by frequency calculations. The vibrational analysis provided thermal enthalpy and entropy corrections at 298 K within the rigid rotor/harmonic oscillator/ideal gas approximation [21]. Thermochemical corrections were scaled by a factor of 0.985, as suggested in (https://comp.chem.umn.edu/freqscale/version3b2.htm, accessed on 25 January 2025). The integration grid was set to ultrafine. The solvation effect was estimated using the implicit solvation model (Conductor-like Polarizable Continuum Model (CPCM)) [26]. For the solution, a correction for the change in the standard state (1 atm→1 mol/L, ΔG°→* = 1.89 kcal/mol) was taken into account. More accurate reference thermochemical calculations were performed using the high-level compound CBS-QB3 method [20].

## 4. Conclusions

The de-alkoxylation condensation of alkoxy-functional silanes in the presence of a ketone was investigated using GC, ^1^H NMR, ^29^Si NMR, and GC-MS. This reaction catalyzed Cp*Ge^+^ B(C_6_F_5_)_4_^−^. Unlike the previously studied analogous de-alkoxylation of model alkoxysilanes (PhMe_2_SiOMe) in the presence of an aldehyde, the acetone-assisted process was not quantitative. Instead, it led to the formation of an equilibrium mixture that contained alkoxysilane, the corresponding disiloxane, a ketal (2,2-dimethoxypropane, DMP), and unreacted acetone. Additionally, small amounts of the intermediate mixed ketal, Me_2_C(OMe)(OSiMe_2_Ph), were detected by ^1^H NMR and GC-MS.

For the ketone-assisted de-alkoxylation reaction to be reversible, the cleavage of the siloxane bond by a ketal molecule in the presence of catalytic amounts of Cp*Ge^+^ B(C_6_F_5_)_4_^−^ must be possible. Indeed, the cleavage of hexamethyldisiloxane (MM) by DMP was confirmed under these conditions. This study was further extended to investigate the reactions of short linear trimethylsilyl-terminated siloxanes (MDM, MD_2_M, MD_3_M, and MD_4_M) with DMP. It was determined that the terminal siloxane bond was the most reactive, which led to the excess formation of methoxy-functional oligosiloxane (MD_x_^OMe^), where x = 0, 1, 2, 3, 4, and Me_3_SiOMe at the onset of the process. Over time, the concentration of this kinetic oligosiloxane product decreased due to subsequent siloxane bond disproportionation reactions and acetone-assisted de-alkoxylation reaction, which were also catalyzed by Cp*Ge^+^ B(C_6_F_5_)_4_^−^. The ^29^Si NMR spectrum of the final mixture indicates the presence of an equilibrium mixture that consisted of trimethylsilyl-terminated and alkoxy-terminated linear oligosiloxanes, as well as Me_3_SiOMe; MM; and cyclic siloxanes, such as D_4_ and D_5_.

Based on experimental results and quantum chemical calculations using DFT and CBS-QB3 methods, a mechanism for the ketone-assisted de-alkoxylation process was proposed. The reaction involves nucleophilic attack by the alkoxysilane on the carbonyl carbon of the ketone, which is activated through interaction with Cp*Ge^+^ B(C_6_F_5_)_4_^−^. The resulting mixed ketal undergoes a subsequent nucleophilic substitution reaction by a second alkoxysilane molecule, leading to the formation of disiloxane and a ketal molecule. The reverse reaction, in which the siloxane oxygen performs a nucleophilic attack on the ketal carbon within the ketal–Cp*Ge^+^ B(C_6_F_5_)_4_^−^ complex, ultimately results in the cleavage of the siloxane bond.

The presented findings indicate that the acetone-assisted de-alkoxylation reaction may have potential applications in controlling the crosslinking of alkoxy-functional siloxane resins, as well as in promoting degradation of the siloxane network upon addition of DMP in the presence of a catalytic amount of Cp*Ge^+^ B(C_6_F_5_)_4_^−^.

## Figures and Tables

**Table 1:** Comparison of DFT and CBS-QB3 values of enthalpy, Gibbs free energy (kcal/mol), and K_eq_ for key reactions and equilibria in the reaction systems studied.

Reaction	Method	ΔH	ΔG	K_eq_
(**A**) EtCHO + 2 Me_3_SiOMe ⇌ EtCH(OMe)_2_ + Me_3_SiOSiMe_3_	DFT ^a^	−14.9	−1.6	15
	CBS-QB3 ^b^	−16.8	−6.6	6 × 10^4^
(**B**) Me_2_CO + 2 Me_3_SiOMe ⇌ Me_2_C(OMe)_2_ + Me_3_SiOSiMe_3_	DFT	−9.2	5.6	8 × 10^−5^
	CBS-QB3	−12.3	−0.9	4.1
(**C**) CpGe^+^-EtCHO + Me_3_SiOMe ⇌ CpGe^+^-MeOSiMe_3_ + EtCHO	DFT	−2.3	−0.6	2.7
	CBS-QB3	−2.9	−1.5	13
(**D**) CpGe^+^-Me_2_CO + Me_3_SiOMe ⇌ CpGe^+^-MeOSiMe_3_ + Me_2_CO	DFT	−0.2	1.0	0.2
	CBS-QB3	−2.7	−1.1	7
(**E**) CpGe^+^-Me_2_C(OMe)_2_ + M_2_ ⇌ CpGe^+^-M_2_ + Me_2_C(OMe)_2_	DFT	5.1	7.5	3.2 × 10^−6^
	CBS-QB3	3.4	4.5	5 × 10^−4^

(a) DFT values given for the gas phase conditions (for consistency with the previous report) [1]. (b) CBS-QB3 values given for CH_2_Cl_2_ solution (for consistency with the experimental conditions).

**Table 2:** Relative DFT enthalpies and Gibbs free energies (kcal/mol) of stationary points along the reaction path in the gas phase for the proposed mechanism (sum of the Gibbs free energies of free reactants arbitrarily set to zero).

Reaction	ΔH_rel_	ΔG_rel_
CpGe^+^ + Me_2_CO + MeOSiMe_3_ (free substrates)	0	0
CpGe^+^–Me_2_CO + MeOSiMe_3_	−18.7	−10.7
CpGe^+^–MeOSiMe_3_ + Me_2_CO	−18.9	−9.7
I	−33.9	−12.1
I-TS	−7.2	20.0
II	−22.4	3.5
CpGe^+^–Me_2_C(OMe)(OSiMe_3_) (III)	−26.8	−0.4
CpGe^+^ + Me_2_C(OMe)(OSiMe_3_) (intermediate)	−3.0	11.5
IV-TS	−26.9	9.1
V	−29.6	4.1
VI-TS	−15.9	25.8
VII	−45.1	−8.4
VIII, CpGe^+^–Me_2_C(OMe)_2_ + Me_3_SiOSiMe_3_	−31.9	−7.2
CpGe^+^ + Me_2_C(OMe)_2_ + Me_3_SiOSiMe_3_ (products)	−9.2	5.6 *

(*) This value was apparently overestimated, as indicated by kinetic data and by calculations using the more accurate CBS-QB3 method. While the computational expense precluded a full CBS-QB3 recalculation of the energy profile, the present DFT values offer a valuable comparison with those previously reported for the analogous reaction with propionaldehyde [1].

## Data Availability

The data presented in this study are available on request from the corresponding author.

## Supplementary Materials

The following supporting information can be downloaded from https://www.mdpi.com/article/10.3390/molecules30143005/s1. Figure S1. GCMS analysis of the reaction mixture that contained 2 equivalents of PhMe_2_SiOMe, 1 equivalent of acetone, and 0.01 equivalents Cp*Ge^+^B(C_6_F_5_)_4_^−^, completed after 3 days of reaction. Figure S2. MS fragmentation pattern of the signals assigned to 2,2-dimethoxypropane (Rt = 3.1 min) and 2-methoxy-2-dimethylphenylsiloxypropane (Rt = 14.9 min). Figure S3. ^29^Si NMR spectrum of the reaction mixture that contained 2 equivalents of PhMe_2_SiOEt, 1 equivalent of acetone, and 0.01 equivalents Cp*Ge^+^B(C_6_F_5_)_4_^−^, recorded after 48 h of reaction. Figure S4. GC/MS analysis with peak assignments of the reaction of [MD_4_M] = 1.2 mol/L with [DMP] = 1.2 mol/L in the presence of [Cp*Ge^+^B(C_6_F_5_)_4_^−^] = 0.0092 mol/L at t = 24 h. Figure S5. Substrate conversion and products formation versus time for the reaction of [MD_3_M] = 1.1 mol/L with [DMP] = 1.1 mol/L in the presence of [Cp*Ge^+^ B(C_6_F_5_)_4_^−^] = 0.0092 mol/L. Solid lines represent MD_X_M oligomers; dashed lines represent MD_x_^OMe^ oligomers. Figure S6. Substrate conversion and products formation versus time for the reaction of [MD_2_M] = 1.2 mol/L with [DMP] = 1.2 mol/L in the presence of [Cp*Ge^+^B(C_6_F_5_)_4_^−^] = 0.0088 mol/L. Solid lines represent MD_X_M oligomers; dashed lines represent MD_x_^OMe^ oligomers. Figure S7. Substrate conversion and products formation versus time for the reaction of [MDM] = 0.98 mol/L with [DMP] = 1.09 mol/L in the presence of [Cp*Ge^+^B(C_6_F_5_)_4_^−^] = 0.0095 mol/L. Solid lines represent MD_X_M oligomers; dashed lines represent MD_x_^OMe^ oligomers. Figure S8. Calculated structures of the stationary points and the transition states of the de-alkoxylation reaction catalyzed by CpGe^+^. Colors of elements: white—H, grey—C, steel grey—Si, darker steel grey—Ge, red—O. Figure S9. Comparison of the ^1^H NMR spectrum of CpGe^+^ B(C_6_F_5_)_4_^−^ with that of a mixture that contained 1 equivalent of CpGe^+^ B(C_6_F_5_)_4_^−^ and 100 equivalents of DMP.
