# Supplementary material for: Ketone-Assisted Alkoxysilane Condensation to Form Siloxane Bonds
Source: Molecules. 2025 Jul 17;30(14):3005. doi: 10.3390/molecules30143005 (PMC12299773; doi:10.3390/molecules30143005)
Supplement: Supplementary file 1 [file molecules-30-03005-s001.zip › molecules-3737890-supplementary.pdf]

Supplemental Information

Ketone-Assisted Alkoxysilane Condensation to Form Siloxane Bonds

Sławomir Rubinsztajn, Marek Cypryk, Jan Kurjata, Małgorzata Kwiatkowska and Urszula Mizerska

Centre of Molecular and Macromolecular Studies of Polish Academy of Sciences, 90-363 Lodz, Poland

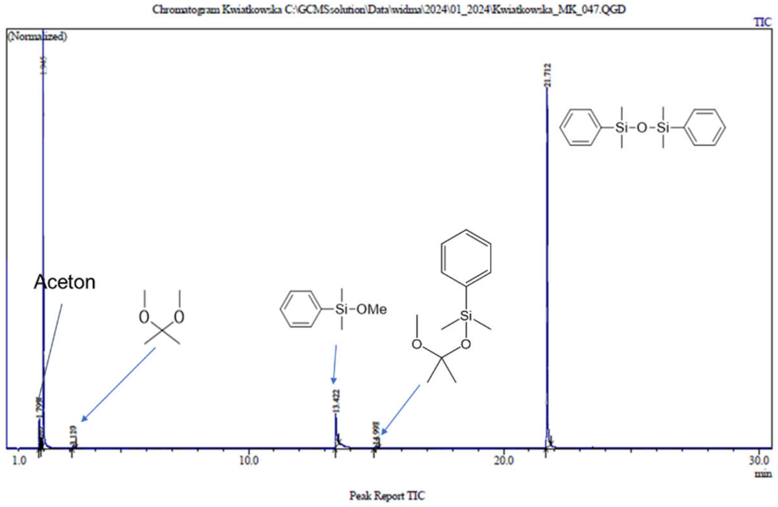

Figure S1. GCMS analysis of the reaction mixture 20 eq. $PhMe_2SiOMe$, 10 eq. of acetone and 1 eq. Cp^*Ge^+ $B(C_6F_5)_4^-$ completed after 3 days of reaction.

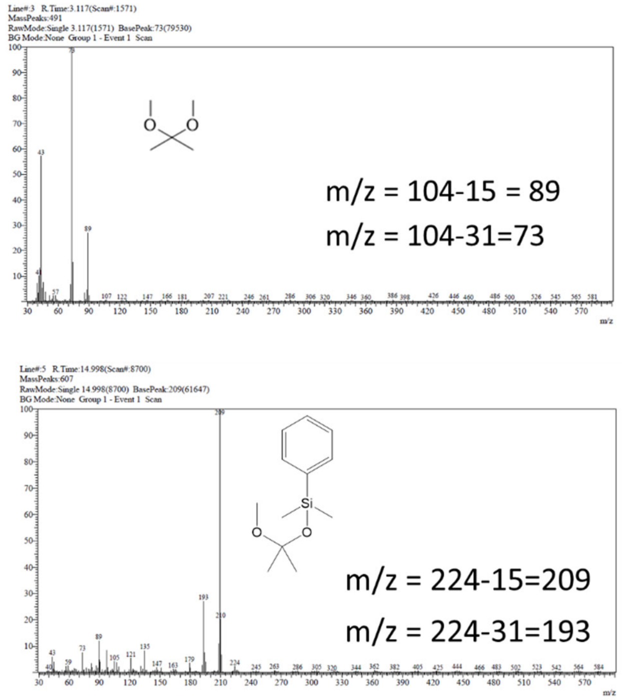

Figure S2. MS fragmentation pattern of the signals assigned to 2,2-dimethoxypropane (Rt= 3.1 min) and 2-methoxy-2-dimethylphenylsiloxypropane (Rt = 14.9 min), Figure S1.

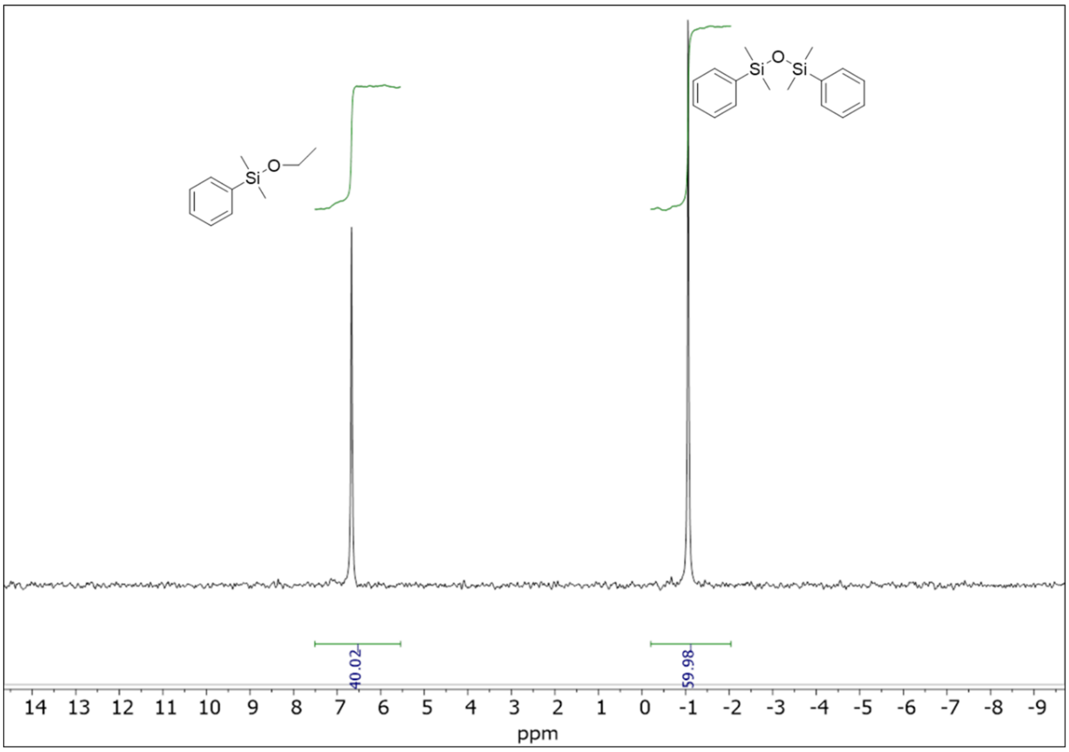

Figure S3. ^{29}Si NMR spectrum of the reaction mixture 20 eq. $PhMe_2SiOEt$, 10 eq. of acetone and 1 eq. $Cp^{*}Ge^{+}\ B(C_6F_5)_4^{-}$ recorded after 48 h of reaction.

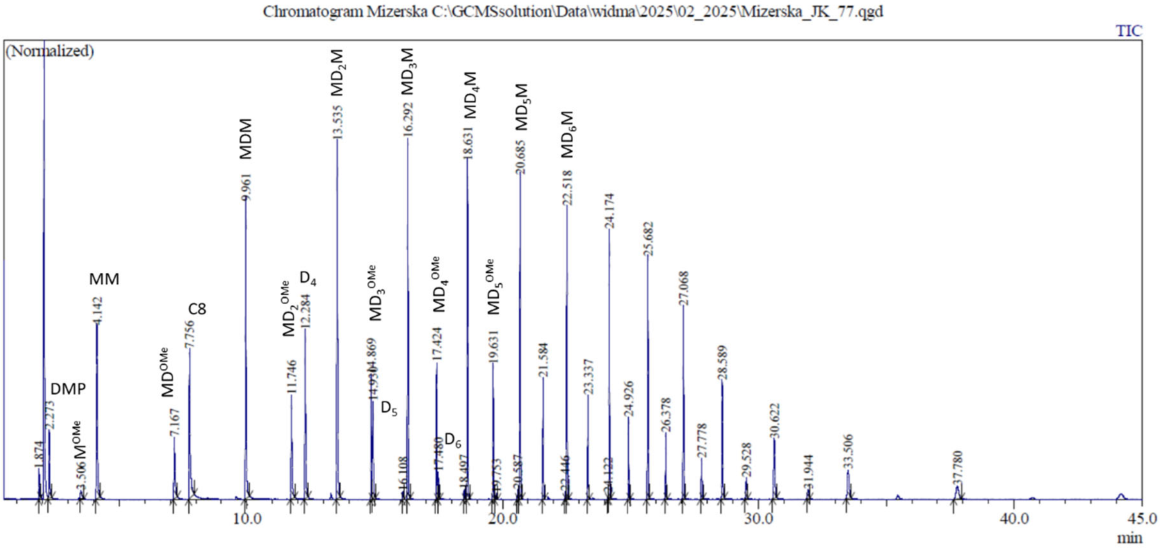

Figure S4. GC/MS analysis with peaks assignments of the reaction of $[MD_4M]$ = 1.2 mol/L with [DMP] = 1.2 mol/L in the presence of $[Cp^{*}Ge^{+}\ B(C_6F_5)_4^{-}]$ = 0.0092 mol/L at t = 24h.

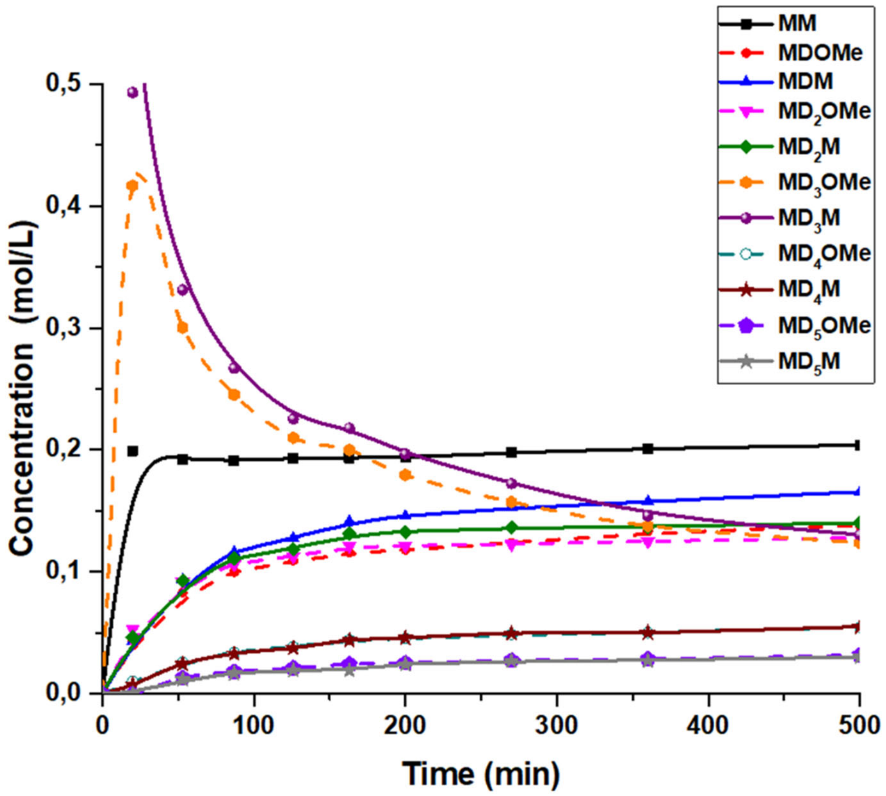

Figure S5. Substrate conversion and products formation versus time *in the reaction of [MD_3M] = 1.*1 *mol/L with [DMP] = 1.1 mol/L in the presence of [Cp^*Ge^+ $B(C_6F_5)_4^-$] = 0.0092 mol/L.*

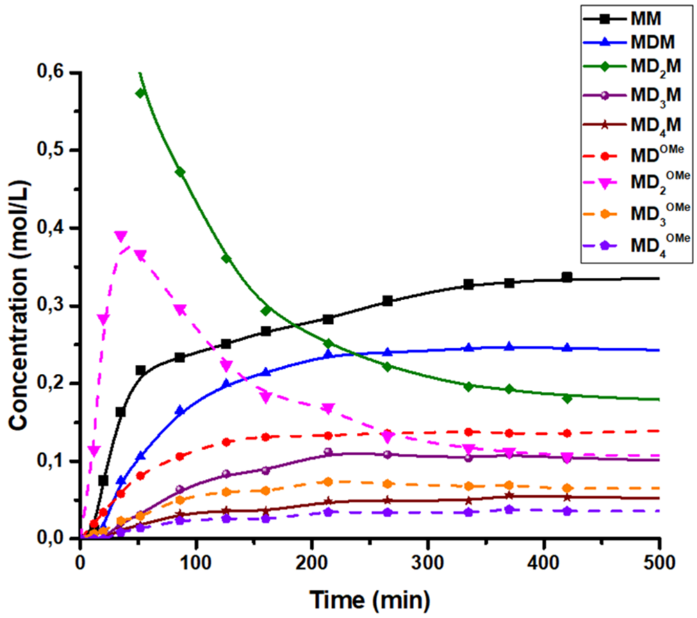

Figure S6. Substrate conversion and products formation versus time *in the reaction of [MD_2M] = 1.2 mol/L with [DMP] = 1.2 mol/L in the presence of [Cp^*Ge^+ $B(C_6F_5)_4^-$] = 0.0088 mol/L.*

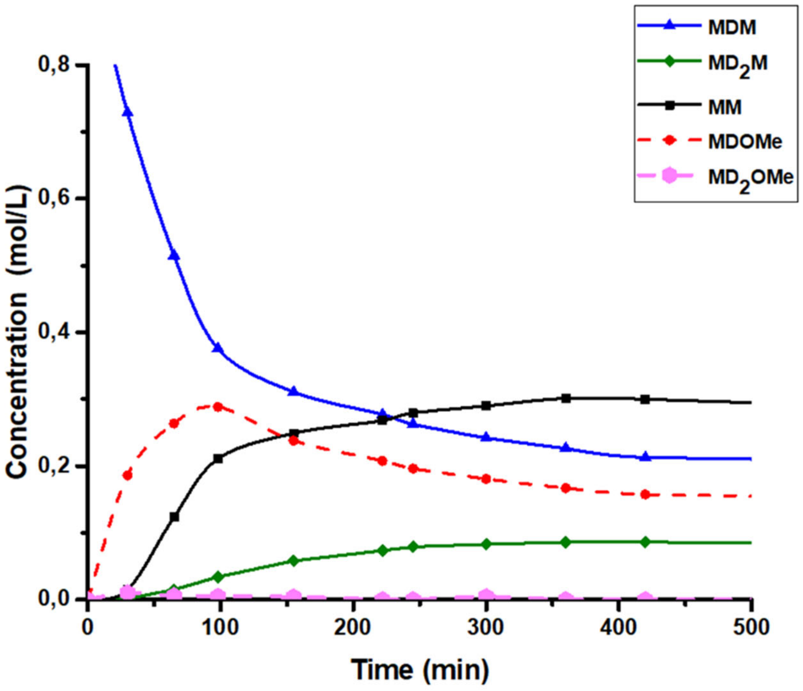

Figure S7. Substrate conversion and products formation versus time in the reaction of [MDM] = 0.98 mol/L with [DMP] = 1.09 mol/L in the presence of [Cp*Ge^{+} B(C_6F_5)$_4^{-}$] = 0.0095 mol/L.

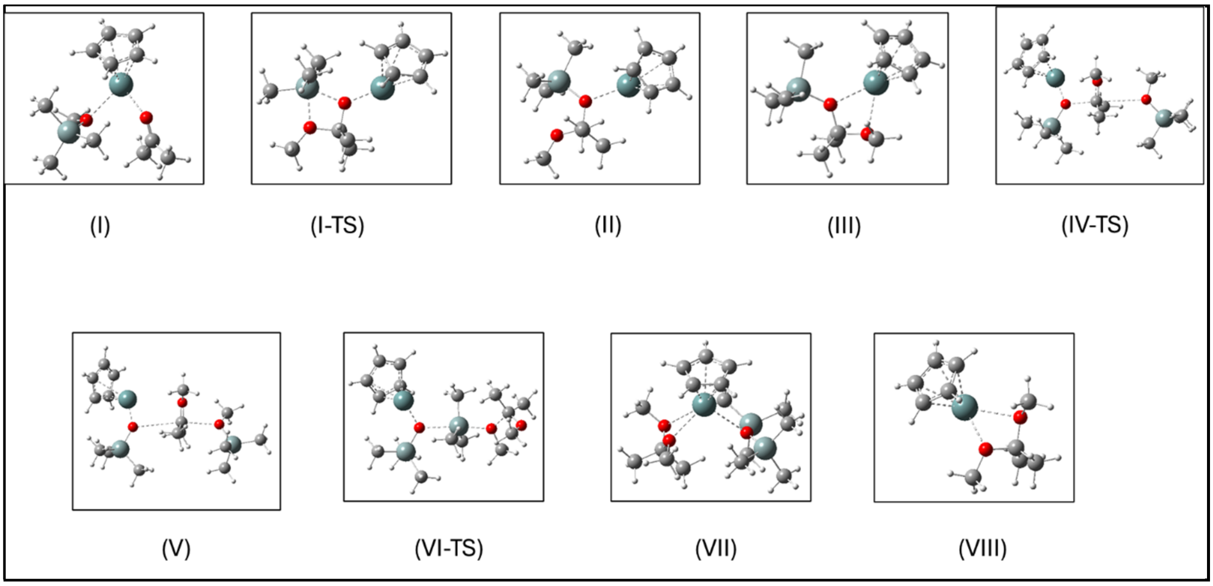

Figure S8. Calculated structures of the stationary points and the transition states of the de-alkoxylation reaction catalyzed by CpGe^{+}. Colors of elements: white – H, grey – C, steel-grey – Si, darker steel grey – Ge, red – O.

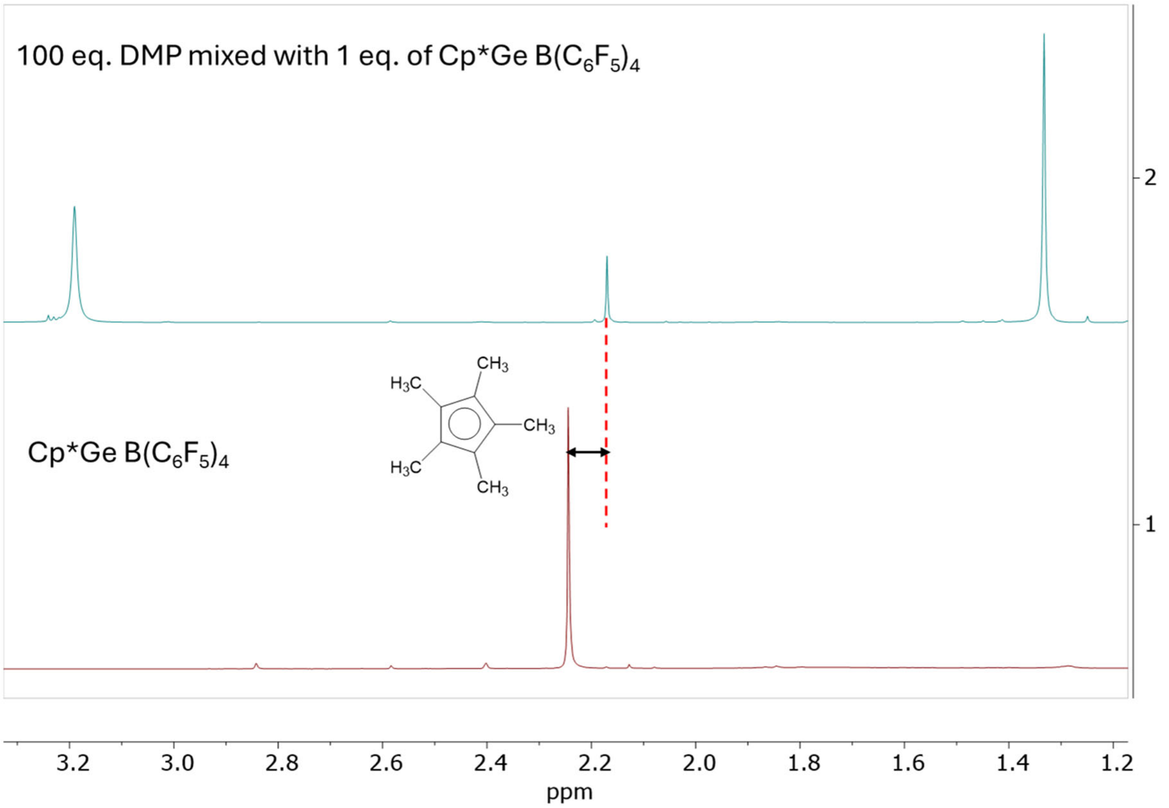

Figure S9. Comparison of the ^{1}H NMR spectrum of $CpGe^+ B(C_6F_5)_4^-$ with that of a mixture containing 1 equivalent of $CpGe^+ B(C_6F_5)_4^-$ and 100 equivalents of DMP.
